# Measurement of Malrotation on Direct Radiography in Pediatric Distal Radius Fractures: Prospective Observational Study

**DOI:** 10.1097/MD.0000000000003569

**Published:** 2016-05-06

**Authors:** Tahir Mutlu Duymus, Serhat Mutlu, Baran Komur, Harun Mutlu, Bulent Yucel, Atilla Sancar Parmaksizoglu

**Affiliations:** From the Department of Orthopaedics, Istanbul Kanuni Sultan Suleyman Training and Research Hospital, Küçükçekmece, Istanbul, Turkey (TMD, SM, BK, BY), and Department of Orthopaedics, Taksim Training and Research Hospital, Gaziosmanpasa, Istanbul, Turkey (HM, ASP).

## Abstract

The aim of this prospective study was to test a mathematical method of measuring the malrotation of pediatric distal radius fractures (PDRFs) from direct radiographs. A total of 70 pediatric patients who presented at the Emergency Department with a distal radius fracture were evaluated. For 38 selected patients conservative treatment for PDRF was planned. Anteroposterior and lateral radiographs were taken of all of the patients for comparison before and after reduction. Radius bone diameters were measured in the coronal and sagittal planes on the healthy and fractured sides. Using the diameter values on the healthy side and the new diameter values on the fractured side in the rotation formula, the degree of malrotation between the fracture ends was calculated. The mean follow-up period was 13.5 months. Patients’ mean age was 10.00 ± 3.19 years (range, 4–12 years). The rotation degree in the sagittal plane significantly differed between the proximal (26.52°±2.84°) and distal fracture ends (20.96°±2.73°) (*P* = 0.001). The rotation degree in the coronal plane significantly differed between the proximal (26.70°±2.38°) and distal fracture ends (20.26°±2.86°) (*P* = 0.001). The net rotation deformity of the fracture line was determined to be 5.55°± 3.54° on lateral radiographs and 5.44°± 3.35° on anteroposterior radiographs, no significant difference was observed between measurements (*P* >0.05). The malrotation deformity in PDRF occurs with greater rotation in the proximal fragment than in the distal fragment. The net rotation deformity created between the fracture ends can be calculated on direct radiographs.

**Level of Evidence:** Diagnostic, Level II

## INTRODUCTION

Pediatric distal radius fractures (PDRFs) are a common occurrence in the pediatric population.^1^ Immediate closed reduction and casting with procedural sedation is the mainstay of management for displaced fractures. Several studies have shown that most cases of PDRFs are treated with closed reduction and heal without complications^2–4^ because they are metaphyseal-region fractures and have a high remodeling capacity. In these fractures, complete bayonet apposition is acceptable, provided angulation and growth remain within appropriate limits.^5^ However, advanced displacement or angular malunion may lead to rotation deformities affecting daily activities.^6^

To evaluate redisplacement, adequate remodeling, normal growth, and rotation defect, repeated radiographs must be taken during follow-up.^7^ Although angulation or displacement measurements can be clearly made on direct anteroposterior and lateral (AP/LAT) radiographs, measurement of malrotation is limited to the subjective evaluation of landmarks on comparative radiographs. The diameter of the distal radial region in the coronal plane is greater than the diameter in the sagittal plane. In this study, we aimed to calculate the rotation deformity of PDRFs in the axial plane using a new method employing the diameter differences on AP/LAT radiographs and the changes in the new diameters that develop following fracture.

## PATIENTS AND METHODS

### Study Design

A total of 70 pediatric patients who presented at the Emergency Department of a large urban teaching hospital with a distal radius fracture were evaluated. The study included a prospective selection of 38 patients with isolated, closed, displaced PDRFs. All of the participants provided written informed consent before this study and the study was approved by the Local Ethics Committee, Taksim Education and Research Hospital, Turkey (ID Number: 43-15).

### Study Setting and Population

The inclusion criteria were children aged 4 to 12 years with a distal radius fracture. Exclusion criteria were children <4 years or >12 years of age, nondisplaced fracture, Salter-Harris III/IV fractures, intra-articular fractures, operative cases, and open fractures. All of the patients were examined, and standard radiographs of the wrist and forearm were taken before reduction.

All the fractures were reduced by an orthopedic surgeon after appropriate analgesia. PDRFs with reduction were treated with an above-the-elbow plaster cast for 6 weeks. Plaster casting was applied with the wrist in full supination and the elbow at 90°. AP/LAT radiographs were taken of both forearms with the patients in a standing position, the elbow at 90°, and the wrist in full supination for measurements. Both forearms and wrists were maintained in the same position symmetrically.

The remanipulation criteria for radius fractures were >20° angulation on the lateral radiograph, >10° angulation on the anteroposterior radiograph, <50% apposition on either radiograph, or >15° change in 1 week on the lateral radiograph. All of the patients were followed up to clinical and radiographic union, with the first radiographs generally taken 7 to 10 days after casting. Follow-up clinic visits took place weekly for the first 4 visits, then at 6 or 8 weeks when the casts were removed. The mean follow-up period was 13.5 months (range, 12–14 months).

### Radiological Measurements

Radiographs taken at initial presentation and after reduction were analyzed for various measurements including the location of the fracture, angulation, and displacement. The coronal and sagittal diameters of the radius were measured on both the healthy and fractured sides. The measurements were made by one orthopedic surgeon (TMD) on the digital radiographs using the standard imaging program at our hospital. The diameters of the proximal and distal fracture ends were measured from the most external edges on the AP and LAT radiographs.

**FIGURE 1:**
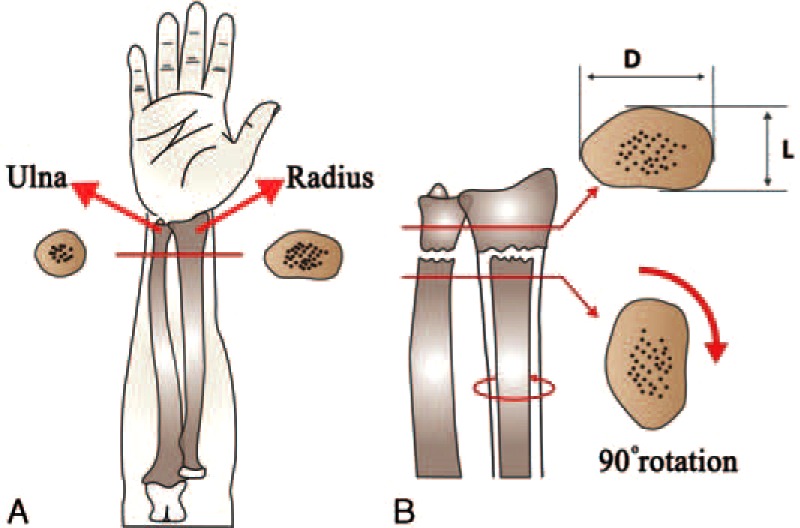
(A**)** The transverse cross-section of the distal radius and ulna. (B) The coronal diameter was labeled as “*D*” and the sagittal diameter as “*L*.” *D*** = **on the AP radiograph of the healthy side, the coronal diameter was labeled as “*D*,” *L*** = **on the LAT radiograph of the healthy side, the sagittal diameter was labeled as “*L*”.

On the AP/LAT radiograph of the healthy side, the coronal diameter was labeled as “*D*” and the sagittal diameter as “*L*”. When 90° rotation was applied to the proximal fragment of the distal radius fracture, the coronal diameter of the radius “*D*” appeared to have changed location toward the sagittal diameter “L” (Figure 1A and B).

**FIGURE 2:**
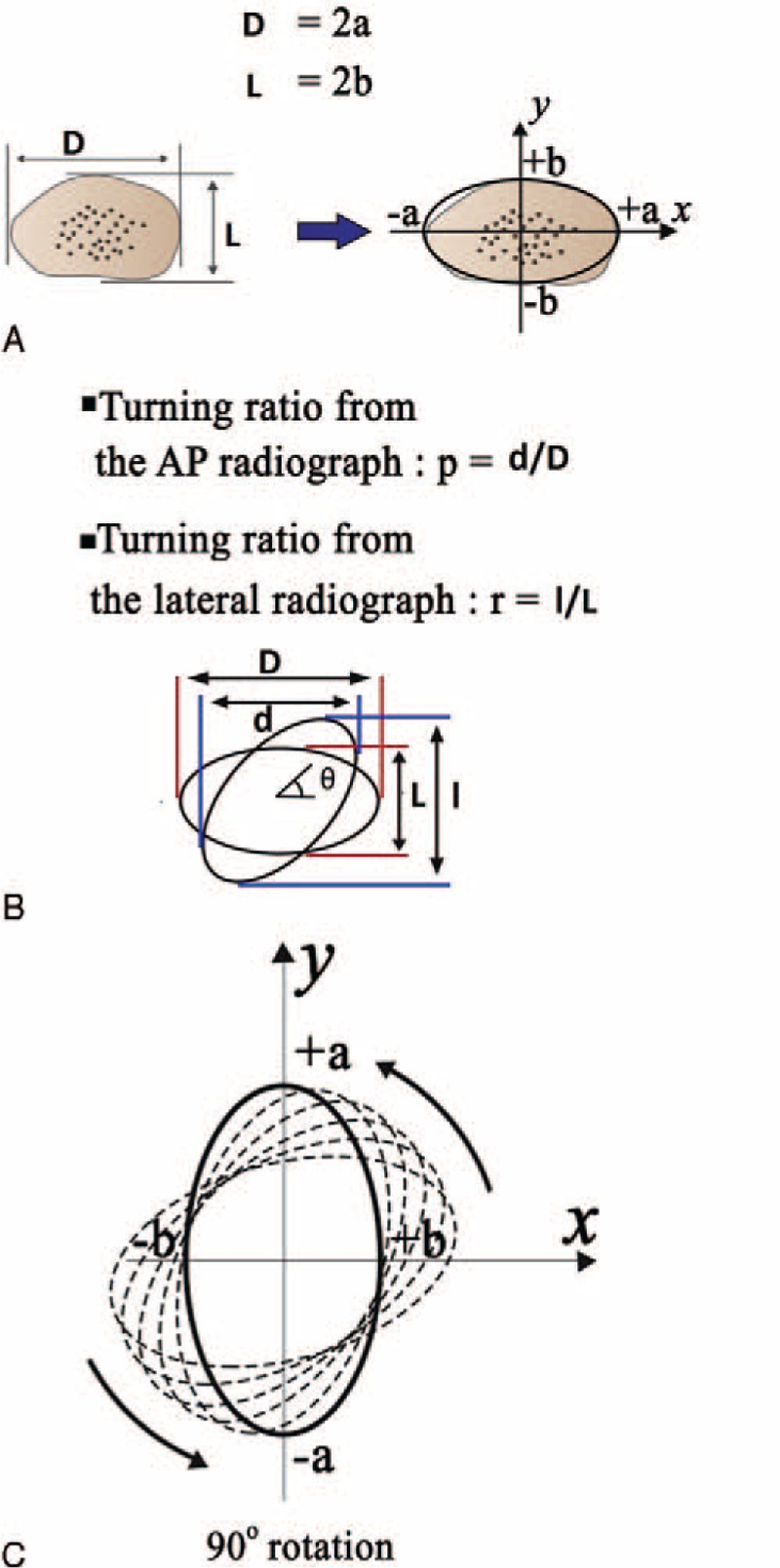
(A) The coronal (*D* = 2*a*) and sagittal (*L* = 2*b*) diameter of the radius. (B) The new diameters after casting were labeled as “*d*” and “*l*.” (C) Ellipsoid movement around the axis of the radius is shown. *a*** = **on the healthy side, the coronal semi-diameter was labeled as “*a*,” AP**-**θ** = **the rotation angle from the AP radiograph, *b*** = **on the healthy side, the sagittal semi-diameter was labeled as “*b*,” *D*** = **on the AP radiograph of the healthy side, the coronal diameter was labeled as “*D*,” *d*** = **the new diameters on the AP radiograph after casting were labeled as “*d,*” *L*** = **on the LAT radiograph of the healthy side, the sagittal diameter was labeled as “*L,*” *l*** = **the new diameters on the lateral radiograph after casting were labeled as “*d*”.

The new diameters on the AP/LAT radiograph after casting were labeled as “*d*” and “*l*” (Figure 2B). As the coronal diameter of the radius on the AP radiograph was greater than the sagittal diameter, an ellipsoid movement around the axis of the bone was apparent (Figure 2C). The rotation defect in the fracture line was calculated with a mathematical formula starting from the diameter values of the healthy side (*D*, *L*) and the new diameter values of the fractured side (*d*, *l*).^8^

### Mathematical Formulas

The coronal diameter of the radius bone of the healthy side on the AP radiograph was defined as “*D*,” and the radius of that semi-diameter as “*a*” (*D* = 2*a*). The sagittal diameter on the lateral radiograph was defined as “*L*,” and the radius of that semi-diameter as “*b*” (*L* = 2*b*) (Figure 2A).

Using the healthy side as the reference, the *a* and *b* radius values of the proximal and distal ends of the fracture line were determined. As shown in Figure 2B, after the fracture, the following parameters were determined:

- The rotation angle occurring in the fracture line “θ” (theta),
- The new diameter value on the lateral radiograph “*l*”
- The new diameter value on the AP radiograph “*d*”
- The turning ratio on the lateral radiograph *r* = *I*/*L*
- The turning ratio on the AP radiograph *p* = *d*/*D*.

**FIGURE 3:**
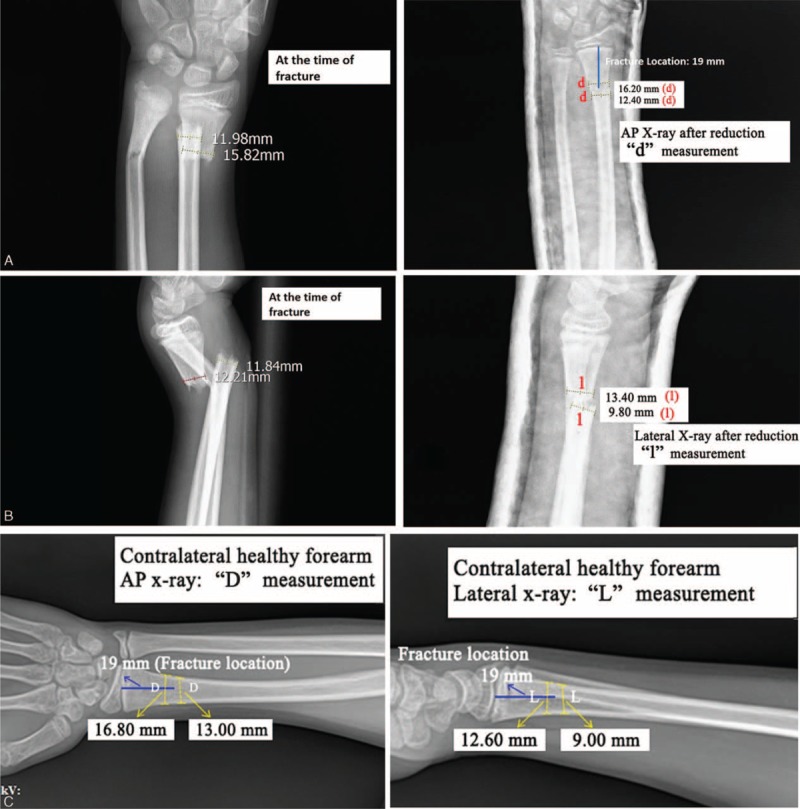
(A-1) AP radiographs at the time of fracture. (A-2) LAT radiographs at the time of fracture. (B-1) AP radiographs and *d*, *l* measurements after reduction in an 11-year-old patient with proximal fragment rotation AP-θ 24.59°, distal fragment AP-θ 23.61°. Rotation defect AP-Δ was 0.99°. (B-2) LAT radiographs and *d*, *l* measurements after reduction in an 11-year-old patient with proximal fragment rotation LAT-θ 24.42°, distal fragment LAT-θ 24.23°. Rotation defect LAT-Δ was 0.19°. (C-1) AP radiographs and *D*,*L* measurements of the contralateral healthy forearm (C-2) LAT radiographs and *D*,*L* measurements of the contralateral healthy forearm in an 11-year-old patient with proximal fragment rotation LAT-θ 24.42°, AP-θ 24.59°, distal fragment LAT-θ 24.23°, and APθ 23.61°. Rotation defect LAT-Δ was 0.19°, and AP-Δ was 0.99°. AP-**Δ** = the rotation defect formed from the difference of the proximal and distal AP-θ, AP**-**θ** = **the rotation angle from the AP radiograph, *D*** = **on the AP radiograph of the healthy side, the coronal diameter was labeled as “*D*,” *d*** = **the new diameters on the AP radiograph after casting were labeled as “*d,*” *L*** = **on the LAT radiograph of the healthy side, the sagittal diameter was labeled as “*L,*” *l*** = **the new diameters on the lateral radiograph after casting were labeled as “*d*,” LAT-**Δ** = the rotation defect formed from the difference of the proximal and distal, LAT**-**θ** = **the rotation angle from the lateral radiograph.

The degree of rotation (θ) was calculated using the rotation formula using the values of the healthy side diameters (*L*, *D*), the radii of those diameters (*a*, *b*), their ratio (*a*/*b*), and the new diameters formed as a result of rotation on the fractured side (*l*, *d*). The degree of rotation can be obtained using one of two different formulas using both AP and LAT images (Figure 3A-1, A-2, B-1, B-2, C-1, C-2):

**Formula 1** for the rotation angle from the lateral radiograph LAT-θ (theta)
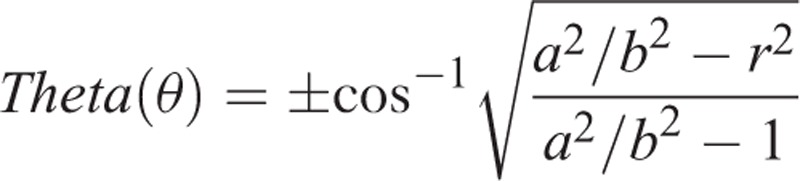


**Formula 2** for the rotation angle from the AP radiograph AP-θ (theta).
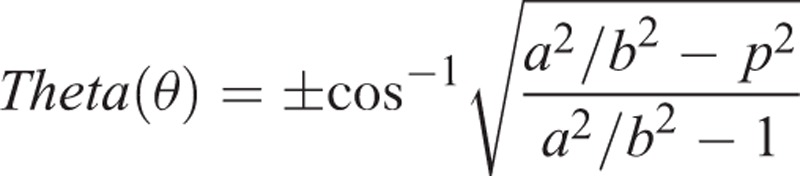


The rotation defect formed from the difference of the proximal and distal AP-θ was defined as AP-Δ (delta), and the rotation defect formed from the difference of the proximal and distal LAT-θ was defined as LAT-Δ

### Clinical Evaluation

All of the patients were clinically examined at 2 and 6 months after the initial trauma. The pronation and supination were graded using a previously employed grading system of excellent, good, fair, and poor.^9^

### Statistical Analysis

SPSS 19.0 software was used for statistical analysis. Categorical variables were described as frequency and percentage. Continuous variables were described as mean, standard deviation, median, minimum, and maximum values. The Shapiro–Wilk test was used to assess the normality of distribution. The Mann–Whitney *U* test was used for independent 2-group comparisons. The relationships among continuous variables were evaluated with Spearman analysis. The Bland–Altman method was used to evaluate the agreement of 2 measurements. In all statistical comparisons, a value of *P* <0.05 was considered statistically significant.

## RESULTS

### Radiological Results

The mean age of 38 patients was 10.00 ± 3.19 years (range, 4–11 years). The distal radius fracture was on the right side in 26 patients and on the left side in 12. The mean fracture location was 21.00 ± 4.44 mm (range, 12–28 mm) from the distal physis line.

The rotation degree in the proximal fragment of the fracture line measured from the LAT radiograph, LAT-θ, was 26.52°±2.84°, and the AP-θ angle from the AP radiograph was 26.70°±2.38°. In the distal fragment of the fracture line, LAT-θ was 20.96°±2.73°, and AP-θ was 20.26°±2.86° **(**Tables 1 and 2**).** The proximal fragment of the fracture line was observed to have undergone more rotation than the distal fragment.

**TABLE 1:**
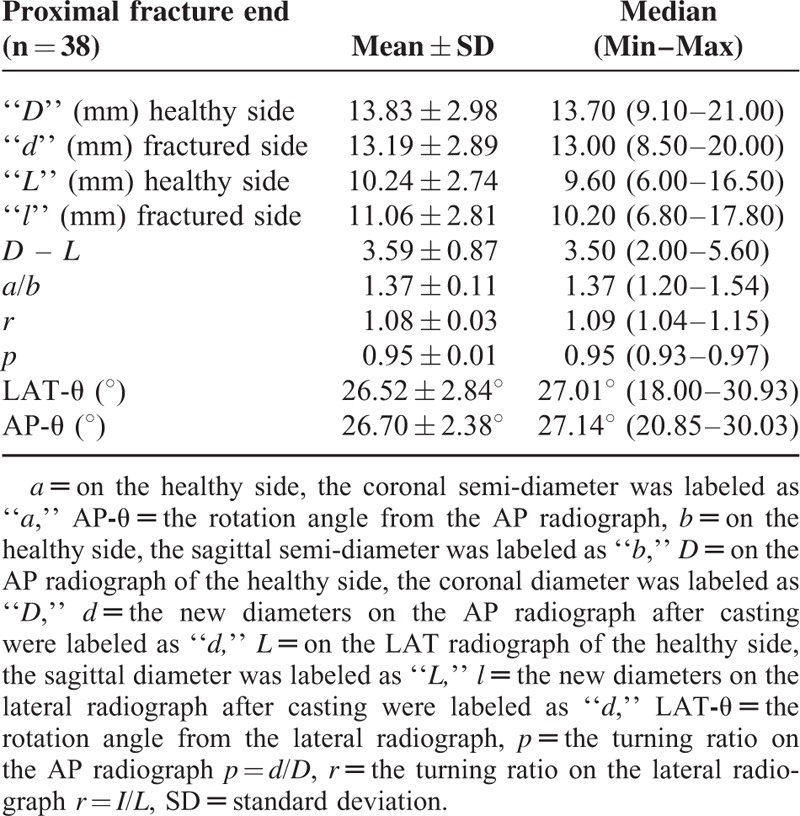
The Comparative Forearm AP/LAT Radiographs With the “*D*” and “*L*” Values, the New Diameter Values of “*d*” and “*l*” Obtained from the AP/LAT Radiographs Taken After Reduction and Casting, and the Formula Components to Calculate the Degree of Rotation in the Proximal Fragments

**TABLE 2:**
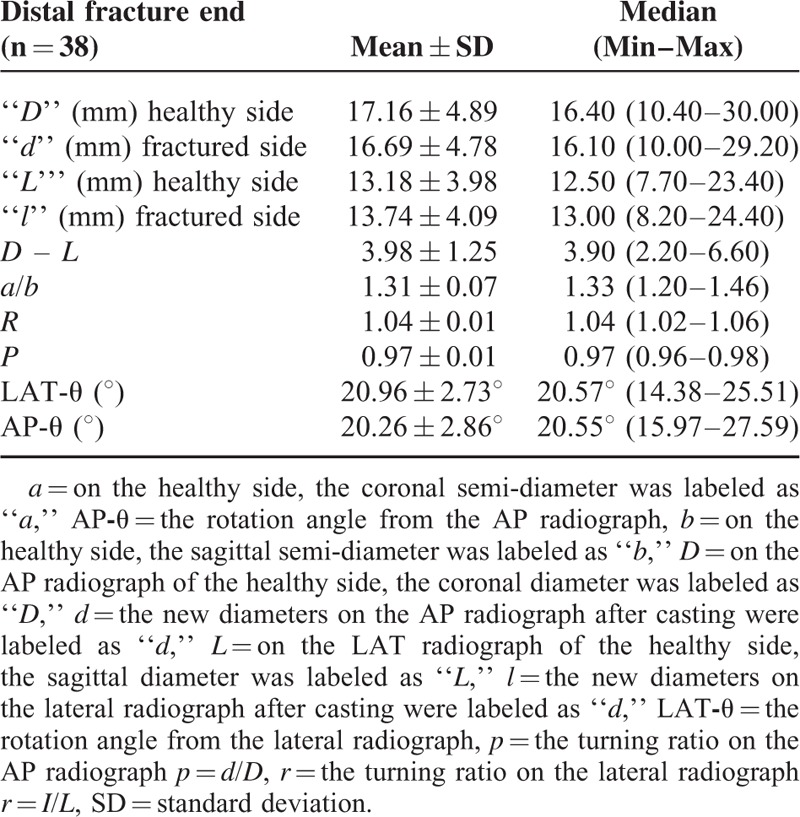
The Comparative Forearm AP/LAT Radiographs with the “*D*” and “*L*” Values, the New Diameter Values of “*d*” and “*l*” Obtained from the AP/LAT Radiographs Taken After Reduction and Casting, and the Formula Components to Calculate the Degree of Rotation in the Distal Fragments

#### Comparison of the LAT-θ and AP-θ Measurements in the Proximal Fracture End

**FIGURE 4:**
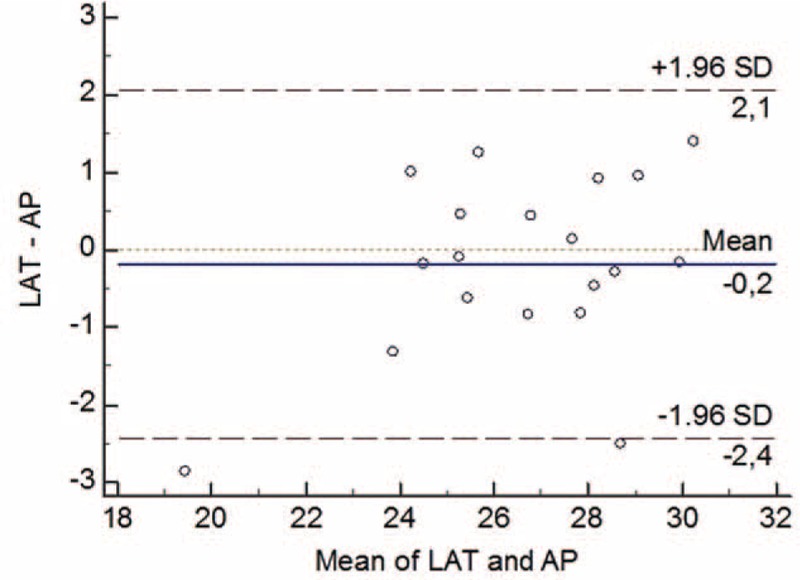
Bland–Altman graph. The graph that indicates concordance the LAT-θ and AP-θ measurements in the proximal fracture end. AP-θ = the rotation angle from the AP radiograph, LAT-θ = the rotation angle from the lateral radiograph.

No significant difference was observed between the LAT-θ and the AP-θ measurements (*P* >0.05). Thus, both methods could be used for the calculation (Table 3, Figure 4).

**TABLE 3:**
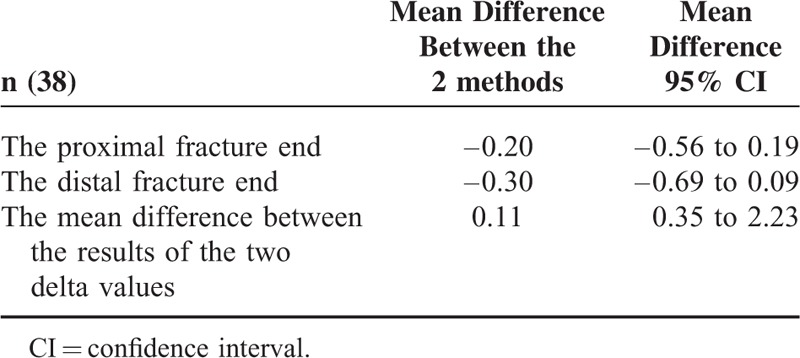
The Mean Difference Between the Results of the Two Theta Values *in the* Proximal and Distal *Fracture End and* the Mean Difference Between the Results of the Two Delta Values

#### Comparison of the LAT-θ and AP-θ Measurements in the Distal Fracture End

**FIGURE 5:**
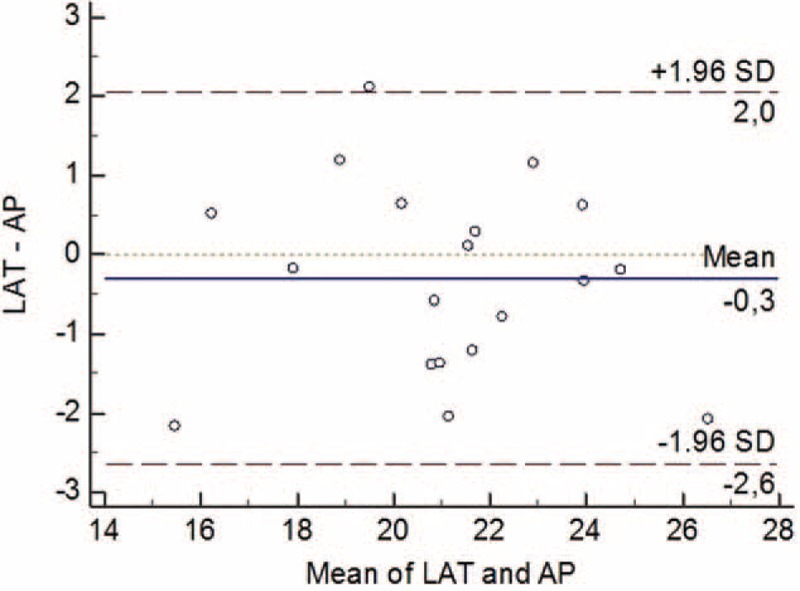
Bland–Altman graph. The graph that indicates concordance the LAT-θ and AP-θ measurements in the distal fracture end. AP-θ = the rotation angle from the AP radiograph, LAT-θ = the rotation angle from the lateral radiograph.

No significant difference was observed between the LAT-θ and the AP-θ measurements (*P* >0.05). Thus, both methods could be used for the calculation (Table 3, Figure 5).

#### Degree of Rotation Occurring in the Proximal and Distal Fracture Ends

In the measurements of the rotation between the proximal and distal fracture ends on the AP and LAT radiographs, statistically significantly more rotation was observed in the proximal fragment than in the distal fragment. In the proximal fragment, both the LAT-θ and the AP-θ values were greater (*P* <0.001∗) (Table 4).

**TABLE 4:**
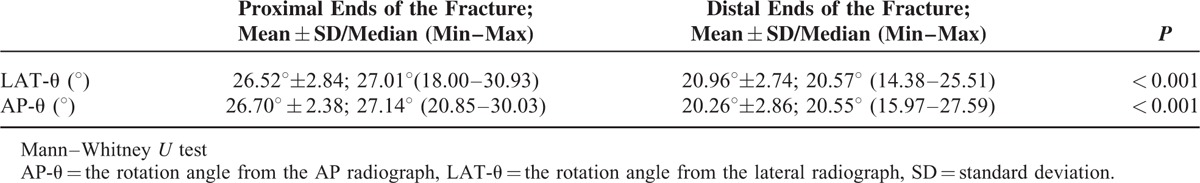
Comparison of the Rotation Degrees in the Proximal and Distal Ends of the Fracture Line

#### Comparison of the AP-Δ (Delta) and LAT-Δ (Delta) Measurements and the Net Rotation Defect in the Proximal and Distal Fragments

**FIGURE 6:**
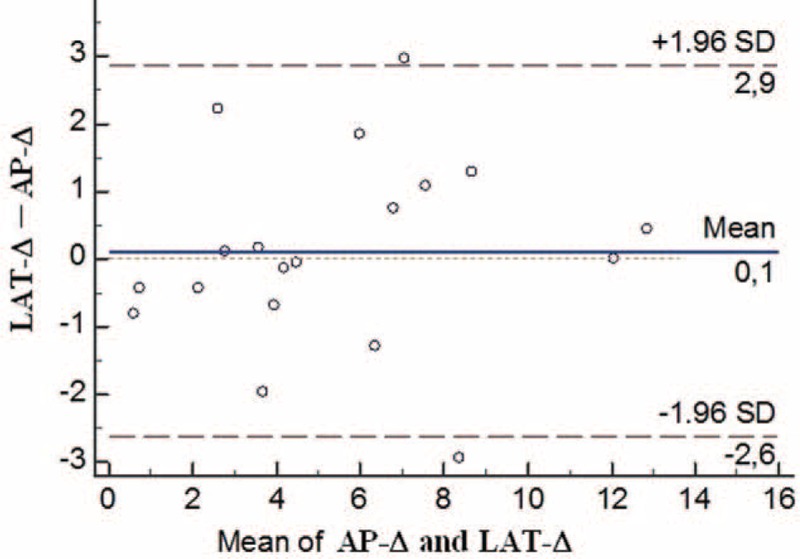
Bland–Altman graph. The graph that indicates concordance the LAT-Δ and AP-Δ measurements. AP-**Δ** = the rotation defect formed from the difference of the proximal and distal AP-θ, LAT-**Δ** = the rotation defect formed from the difference of the proximal and distal.

No significant difference was observed between the AP-Δ and the LAT-Δ measurements (*P* = 0.624). As the methods for calculation are similar, either could be used (Table 3, Figure 6). The differences between the rotation degrees in the proximal and distal ends of the fracture line were calculated, and the net rotation defect formed in the forearm was measured. LAT-Δ was determined as 5.55° ± 3.54°and AP-Δ as 5.44° ± 3.35° (Table 5).

**TABLE 5:**
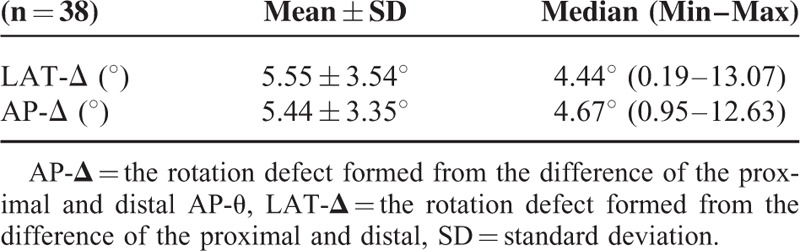
LAT-Δ versus AP-Δ Values on the AP/LAT Radiographs

### Clinical Results

The mean follow-up period was 13.5 months (range, 12–14 months). All fractures healed and achieved full wrist flexion and extension and forearm rotation with excellent results. The mean time to achieve full wrist range of motion after immobilization was 3 weeks (range, 2–5 weeks).

## DISCUSSION

Following PDRFs, the most frequently seen causes of malunion are volar tilt loss in the sagittal plane, ulnar inclination loss in the frontal plane, and transverse section rotation deformity.^10–12^ Union in angular malalignment, translation, or incorrect rotation may lead to restricted forearm functionality.^13–15^ Therefore, to check reduction loss after casting, direct radiographs must be taken during follow-up. Although the amounts of translation or angular deformity in the coronal and sagittal planes of fractures in the long bones are easily determined on direct radiographs, rotational deformities are difficult to determine in the transverse plane.^16^ Although a rotation defect or its degree can be suspected from radiographs, clear measurement and quantitative diagnosis remain the subjects of ongoing research. Very few clinical and experimental studies have investigated the definition of rotational deformities created as a result of forearm or distal radius fractures. Creasman et al recommended that when rotation is suspected, comparative radiographs should be examined.^17^ Evans also reported that bone diameters in the fragment ends could be compared or the positions of the radial tuberosity and radial styloid could be considered on comparative radiographs after reduction.^18^

The degree of malrotation can be determined indirectly on computed tomography (CT) by measuring the rotational defect in the transverse plane and comparing it with the healthy side.^19,20^ However, the application of this method is difficult, especially in the pediatric age group. Furthermore, its costs are high and the workload of the Emergency Department may be increased. This assessment could also lead to ethical problems regarding exposure to intense radiation.

Although malrotation is a question in the assessment of long-bone fractures, in some studies, evaluation has only been made clinically, and rotation angles have not been reported.^21^ This issue indicates the need for a simpler method of malrotation measurement that can be performed after casting. Direct radiography would be ideal for this aim because it is widely available, easy to perform, causes less radiation than CT, and is less expensive.

Previous studies related to the measurement of the degree of malrotation in long-bone (humerus and femur) fractures on direct radiographs have been reported.^22,23^ However, the methods in those studies were developed for diaphyseal fractures of the long bones, whereas the new method reported in the present study was used for fractures in the metaphyseal region. Using this method for the metaphyseal region, a formula was obtained on the basis that the bone is wider in the coronal plane than in the sagittal plane. The diaphyseal sections of tubular bones take on a form close to a cylindrical structure around their own axis, whereas in the metaphyseal region, the bone structure is ellipsoid around its own axis. Because of this ellipsoid property, we concluded that the degree of rotation can be calculated from the changes in diameter occurring after a fracture that can be seen on AP/LAT radiographs.

In the present study, when the coronal and sagittal axial diameters (*D*, *L*) measured on the AP and LAT radiographs of the distal radius were evaluated, the diameter in the coronal plane was found to be greater than the diameter in the sagittal plane. The significant level of difference in the diameters in the coronal and sagittal plane in the fracture ends (*D* – *L*) or the diameter ratio of >1 in the coronal and sagittal plane (*a*/*b*) is because of the ellipsoid shape of the axial cross-section in the metaphyseal area (Tables 1 and 2). Taking this diameter difference and ellipsoid shape as the starting point, the mathematical formula developed by Stokes was applied in this study.^8^ As a result of this formula, it appeared that the proximal and distal fracture ends rotated in the same direction, and the proximal end underwent more rotation than the distal end. If the two fracture ends underwent rotation in opposite directions, the patient would clinically develop a severe supination/pronation defect. In an experimental study by Dumont et al, severe restriction of supination or pronation was reported to occur particularly in cases of reverse direction of rotational malalignment.^24^ In another experimental study, Kasten et al reported that torsional defects > 30° led to significant loss in pronation or supination.^25^ In the clinical results of the present study, no restriction of supination or pronation was observed in any patient, which supports the view that the fracture ends undergo rotation in the same direction. However, the slight possibility of a rotation defect developing in distal radius fractures compared with diaphyseal area fractures could be a criticism weakening the strength of the hypothesis of this study. Fractures in the distal metaphyseal area have a higher remodeling capacity as they are closer to the growth plate.^26^ Although this difference adds a critical dimension to the study hypothesis, it can be considered of no significance when rotation is in the same direction. The hypothesis that the fracture ends are exposed to rotation in the same direction could be supported by a new study correlated to CT or a sawbone model study. However, in the experimental study have reported that this method may be used.^8^

No difference in clinical results has been reported between above-the-elbow and below-the-elbow casting methods applied following the reduction of displaced PDRFs.^27,28^ In the present study, above-the-elbow casting with the wrist in full supination and the elbow joint at 90° was preferred for all of the patients. Thus, the collection of radiographs was standardized across all of the patients. We speculated that below-the-elbow plaster casting, which allows forearm supination and pronation, could possibly lead to incorrect measurements. In addition, comparison of the measurements of the fractured side with the healthy side with the same symmetrical position of both forearms on the AP/LAT radiographs was considered to be very important in obtaining accurate results.

With this method, a reliable imaging technique is very important to the achievement of high accuracy. Digital radiographs and a magnification program were used in the present study, along with traditional plaster casting materials. The reliability of manual and digital radiographic measurement techniques have been reported to show no significant difference.^29^ However, the ease of analysis and magnification quality of digital radiography can be considered to provide more accurate and successful measurements. In the selection of casting material, fiberglass casting provides a clearer image than traditional plaster casting.^30^ However, as the fiberglass material is not widely available in our country, it was not used in the present study. Limitations of this study include the sensitivity of the measurement required and the difficulties of using the mathematical formula. However, with the support of computing technology, the formula could be used very easily through the development of a simple application that can be installed on smart phones or as an additional practical feature that could be added to radiological imaging programs.

In conclusion, the results of this study indicate that on the basis of the greater thickness of the distal metaphyseal region in the coronal plane than in the sagittal plane, the rotation defect of fractures in the metaphyseal region can be calculated with a mathematical formula on direct radiographs. This method may also be applicable to metaphyseal fractures in the other long bones. However, further clinical studies comparing this method with CT measurements are required to confirm its usefulness.
